# Competition Rather Than Observation and Cooperation Facilitates Optimal Motor Planning

**DOI:** 10.3389/fspor.2021.637225

**Published:** 2021-02-26

**Authors:** Mamoru Tanae, Keiji Ota, Ken Takiyama

**Affiliations:** ^1^Department of Electrical and Electronic Engineering, Tokyo University of Agriculture and Technology, Tokyo, Japan; ^2^Department of Psychology, New York University, New York, NY, United States; ^3^Center for Neural Science, New York University, New York, NY, United States

**Keywords:** motor uncertainty, motor decision-making, risk-sensitivity, Bayesian decision theory, aim point

## Abstract

Humans tend to select motor planning with a high reward and low success compared with motor planning, which has a small reward and high success rate. Previous studies have shown such a risk-seeking property in motor decision tasks. However, it is unclear how to facilitate a shift from risk-seeking to optimal motor planning that maximizes the expected reward. Here, we investigate the effect of interacting with virtual partners/opponents on motor plans since interpersonal interaction has a powerful influence on human perception, action, and cognition. This study compared three types of interactions (competition, cooperation, and observation) and two types of virtual partners/opponents (those engaged in optimal motor planning and those engaged in risk-averse motor planning). As reported in previous studies, the participants took a risky aim point when they performed a motor decision task alone. However, we found that the participant's aim point was significantly modulated when they performed the same task while competing with a risk-averse opponent (*p* = 0.018) and that there was no significant difference from the optimal aim point (*p* = 0.63). No significant modulation in the aim points was observed during the cooperation and observation tasks. These results highlight the importance of competition for modulating suboptimal decision-making and optimizing motor performance.

## Introduction

Despite the importance of optimal motor planning, humans select a suboptimal motor plan in various tasks involving spatial (Wu et al., 2009; Nagengast et al., 2010, 2011a; O'Brien and Ahmed, 2013; Ota et al., 2019b), timing (Ota et al., 2015, 2016, 2019a; Onagawa et al., 2019), and spatiotemporal (Nagengast et al., 2011b) control. A suboptimal motor plan, for example, can be exemplified as aiming at a spot near the goal post in football's penalty kicks. Such an aim point might succeed if lucky, but it also increases the probability of kicking outside of the goal post. Throughout this study, we use the term optimal motor planning as the plan that maximizes the expected reward.

We can qualitatively evaluate the optimal and risk-neutral motor planning given the variance in motor performance (Berger, 1985; Trommershäuser et al., 2008). Compared with optimal motor planning, we refer to risk-seeking as the choice preference for a high reward with a high probability of failure, whereas we refer to risk aversion as the choice preference for a small reward with a low probability of failure (Wu et al., 2009). Both risk-seeking and risk-averse strategies are suboptimal and decrease the expected reward by frequently incurring failure or earning small rewards (Ota et al., 2015, 2016).

Previous studies have elucidated the risk-seeking property inherent in motor planning (Wu et al., 2009; Nagengast et al., 2010, 2011a,b; O'Brien and Ahmed, 2013; Ota et al., 2015, 2016; Onagawa et al., 2019). Nevertheless, few studies have investigated effective ways to improve this property and facilitate optimal motor planning. One possible way to modulate motor planning is interactions with others. Previous studies on interpersonal interaction have found that competition subconsciously changes our motor action (Naber et al., 2013; Varlet and Richardson, 2015; Vaziri-Pashkam et al., 2017). In a task in which two participants compete to touch a target the fastest, participants' reaction times are synchronized, although such synchronization results in a lower probability of winning (Naber et al., 2013). The synchronization of motor patterns is observed in competitions not only in laboratory experiments (Vaziri-Pashkam et al., 2017) but also in the Olympic Games (Varlet and Richardson, 2015).

In addition to competition, cooperation with others modulates human motor action (Richardson et al., 2007; Peng and Hsieh, 2012; Ganesh et al., 2014; Ikegami and Ganesh, 2014). Both competition and cooperation increase the number of effortful actions relative to a task without others (Peng and Hsieh, 2012). Moreover, the observation of others' actions induces synchronization. When two people sit side-by-side in rocking chairs, the movement phase angles between the two rocking chairs can be synchronized (Richardson et al., 2007).

These findings raise the possibility that competing with, cooperating with, or observing other people influences human motor planning under risk. Our earlier study in effect showed that competition with a risk-averse opponent can lead suboptimal motor planning to be optimal (Ota et al., 2020). The aim of this study is to follow up on the possibility that cooperation and observation can also modulate suboptimal motor planning. To address this question, we recruited six experimental groups by combining three types of interactions with two types of virtual partners/opponents (risk-neutral and risk-averse partners/opponents). We used virtual partners or virtual opponents since we can arbitrarily manipulate their properties. For a control group, we also added the individual group where any partners/opponents were not involved in our task. A comparison among seven experimental groups allows us to investigate which types of interpersonal interactions are effective in modulating a risky motor plan.

Understanding how to achieve the best performance is an essential question in movement science. Many world-leading athletes attempt to exhibit their best performance in the Olympic Games. To do so, one should consider what the optimal motor plan is (for example, where is the best aim point for a baseball pitch or tennis serve?). This study may provide a clue on the question of how (competition, cooperation, or observation) athletes should interact with other athletes for their decision-making training.

## Methods

### Participants

We recruited 42 healthy adults (32 males; 20 ± 1.7 years) in this study. The participants provided written informed consent before the experiment. This study was approved by the ethics committees of the Tokyo University of Agriculture and Technology and was performed in accordance with the relevant guidelines (No. 29-36) and regulations in the Declaration of Helsinki 2013. We describe the sample size below.

### Experimental Setup and Gain Function

**Figure 1:**
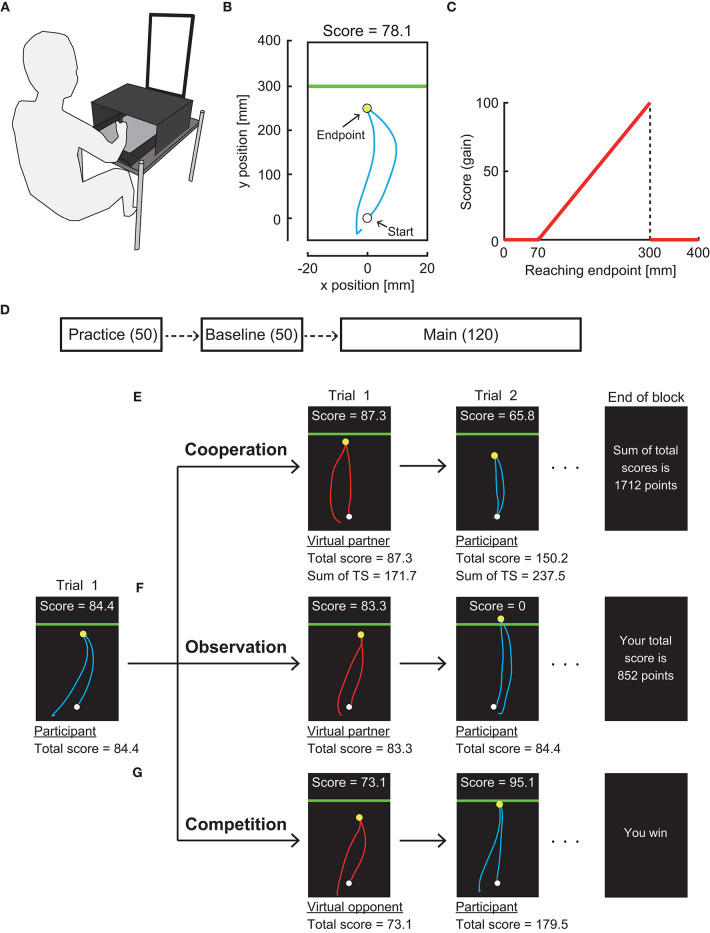
Experimental setting. **(A)** Apparatus. Participants made a quick out-and-back reaching movement on a pen tablet. Stimuli were presented on a vertical display. **(B)** Reaching trajectory in a trial denoted by a cyan curve. A horizontal green line (penalty boundary) was set 30 cm forward from the start position. **(C)** Gain function. Participants obtained a score following this asymmetric gain function. **(D)** Experimental protocol. The participants first practiced the reaching task for 50 trials. They then performed the task under the gain function applied without any partner or opponent (baseline). In the main experimental session, the participants performed either individual, cooperation, observation, or competition task for 12 blocks of 10 trials. **(E)** Cooperation task. **(F)** Observation task. **(G)** Competition task. The participants alternated with the virtual partner or opponent in making a reaching movement.

We used a pen tablet to measure the arm-reaching movement (Wacom, Intuos 4 Extra Large; workspace: 488 × 305 mm). The participants were seated on a chair, and they were instructed to make a quick out-and-back reaching movement while holding the pen on a pen tablet (Figures 1A,B). The position of the digitized pen was sampled at ~144 Hz and was transformed to the position of a cursor on a vertical display (Asus, VG-248QE, 24 inches) at a refresh rate of ~144 Hz. All stimuli were controlled using the Psychophysics Toolbox (Brainard, 1997; Pelli, 1997).

The participants first moved a blue cursor (radius: 0.3 cm) to a white starting position (radius: 0.4 cm) on the display. A green boundary line then appeared 30 cm forward from the starting position, indicating a go signal. The participants rapidly moved the cursor forward and returned it below the starting position. The maximum amplitude of the reaching movement in the sagittal plane (*y*-position) was defined as the endpoint in each trial and marked as a yellow circle (radius: 0.3 cm). If the participants did not return the cursor within 600 ms, no endpoint was shown, and a warning message stated “Time-out. More quickly!” was presented. After a feedback period of 2 s, the participants proceeded to the next trial.

In each trial, the participants obtained a score depending on their reaching endpoint. We used an asymmetric gain function (Figure 1C) in which risk-seeking motor planning has been confirmed (O'Brien and Ahmed, 2013; Ota et al., 2016). The closer the endpoint was to a green boundary line, the higher the score was. If the endpoint fell above the green line, no score was attributed (i.e., mistrial). The maximum score was obtained if the endpoint fell on the line. No score was assigned if the endpoint was less than 7 cm from a starting point, but no such trial was observed (see Ota et al., 2020 for further details of our experimental setup).

### Experimental Protocol and Task

There were three experimental sessions in the following order: practice, baseline, and main session (Figure 1D). First, the practice session of 50 trials was provided to let the participants be familiar with the reaching movement. From the baseline session, the gain function was applied. In the baseline session, there were 5 blocks of 10 trials. In each block, the participants were instructed to maximize the total score of 10 trials while performing the reaching movement alone. The score for a trial and total score were displayed along with the endpoint feedback. There were 12 blocks of 10 trials in the main session. In this session, we randomly assigned one of seven experimental tasks to each participant (i.e., between-subject design). There were (1) individual task, (2–3) competitive tasks with a risk-averse or a risk-neutral virtual opponent, (4–5) cooperation tasks with a risk-averse or risk-neutral virtual partner, and (6–7) observation tasks with a risk-averse or risk-neutral virtual partner.

In the individual task, the participants were instructed to maximize the total score in each block as they did so in the baseline session. In the other six experimental tasks, each trial alternated between the participant's turn and the virtual partner/opponent's turn (Figures 1E–G). That is, after the feedback period in the participant's turn, a red cursor (radius: 0.3 cm) was shown on the display as a start signal of the virtual partner/opponent's turn. A movement trajectory of the red cursor was then automatically manipulated based on prerecorded sample trajectories made by the experimenter. Each movement endpoint was determined by the preprogrammed algorithm described below (see section Manipulation of Virtual Partner and Opponent). The endpoint was marked as a yellow circle along with feedback on the score for a trial and the total score. After this feedback period of 2 s, the turn was switched to the participants.

Instructions differed in three types of interactions. In the competitive task, the participants were asked to achieve a higher total score than their virtual opponents at the end of each experimental block (10 trials). At the end of each block, a message stated “You win” or “You lose” was displayed (Figure 1G). In the observation task, the participants were shown the movement trajectory, movement endpoint, score in a trial, and total score of the virtual partner as in the competitive task. However, the participants were instructed to maximize their own total score. At the end of each block, the participant's total score was shown (Figure 1F). In the cooperation task, the experimental setup was the same as those two tasks, but the participants were instructed to maximize the sum of their and their virtual partner's total scores. The sum of total scores was displayed at the end of each block (Figure 1E).

### Risk Sensitivity

When the trial-to-trial fluctuation of the movement endpoint is modeled as a Gaussian function $N\left(x|\mu ,\sigma^{2}\right)$, the expected gain *G*(μ) for a particular planned movement endpoint μ is defined as

(1) $$\begin{array}{l} \begin{array}{c} G\left(\mu \right)=\int_{-\infty }^{\infty }f\left(x\right)N\left(x|\mu ,\sigma^{2}\right)dx, \end{array} \end{array}$$

where *x* is the actual movement endpoint, σ is the standard deviation of the movement endpoint, and *f*(*x*) is a gain function (Berger, 1985; Trommershäuser et al., 2008). One assumption behind this calculation is that the participant plans the true aim point μ (unobservable variable), while the actual movement endpoint *x* can vary in every trial due to inherent noise in the motor system. Since *x* follows a Gaussian distribution (Trommershäuser et al., 2008), we estimated the participant's aim point μ by taking the average movement endpoint in each block.

Although the observed movement endpoint *x* fluctuates, it is theoretically possible to calculate the expected gain by defining a probabilistic model of *x* and averaging the gain across all possible *x* values. Because the probabilistic model of the movement endpoint approximates a Gaussian distribution (Trommershäuser et al., 2008), we model the expected gain using Equation (1). The other assumption behind this model is that an aim point μ is constant within each block. A previous study shows contradictory evidence to this assumption, that is, an aim point can be corrected in the absence of perturbations (van Beers, 2009). The change in aim points might increase the movement variability σ. However, in our previous study (Ota et al., 2020), we validated that this is not the case. We found that there was no significant difference in the movement variabilities between the individual task where participants could update their aim point and the fixed target condition where the target point was fixed at the point where participants aimed in the individual task. Therefore, whether participants might or might not change their aim point would not jeopardize our argument.

Although it is possible to compute the expected gain with a general form of *f*(*x*) by using a numerical or Monte Carlo integral, Equation (1) is analytically tractable in the current setting or in the case when *f*(*x*) is a piecewise linear function (see Supplementary Material). After calculating *G*(μ) by changing the value of μ, we can find the optimal aim point to maximize the expected gain: $\mu^{*}=argmax_{\mu }G\left(\mu \right)$. Thus, we can discuss the participant's risk sensitivity by comparing the participant's aim point and μ^*^. When the participant's aim point is larger than μ^*^, their motor plan can be considered risk-seeking (O'Brien and Ahmed, 2013; Ota et al., 2016). When the participant's aim point is smaller than μ^*^, their motor plan can be considered risk-averse (O'Brien and Ahmed, 2013; Ota et al., 2016). When there is no significant difference, the motor plan is optimal or risk-neutral. The risk-seeking strategy indicates that participants seek a high one-trial gain with a high probability of failure. The risk-averse strategy indicates that participants seek a low one-trial reward and to avoid a high probability of failure. Both strategies are suboptimal in terms of maximizing the expected gain.

To determine the optimal aim point in each block, we estimated the participant's movement variance σ^2^. In our previous study (Ota et al., 2020), we estimated the movement variance based on the variability of the endpoint in each block (i.e., 10 trials). To obtain a more reliable measurement, this study utilized the variability of endpoints in the past 40 trials. As a result, the risk sensitivity for the 1st to 4th block was not evaluated.

### Manipulation of Virtual Partner and Opponent

We used a virtual partner or opponent in this study. The advantages of using a virtual partner/opponent are as follows: First, we can arbitrarily manipulate their risk sensitivity. Second, we can set their movement accuracy to the same degree as the participant's movement accuracy. These advantages enable us to examine the influence of interaction type while changing the partner's/opponent's risk sensitivity and controlling their movement accuracy, which is a possible confounding factor.

**Figure 2:**
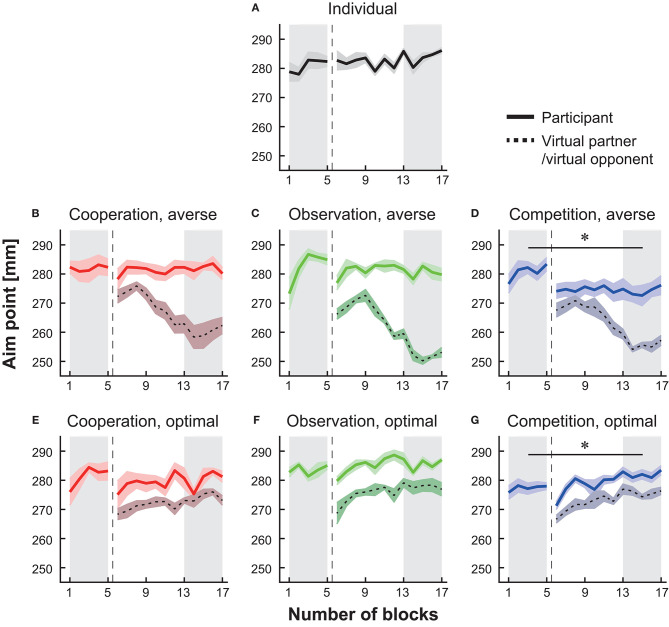
Block-to-block variation in the mean endpoint (i.e., aim point). The data from the baseline to the main session are shown in the individual group **(A)**, in the cooperation groups with a risk-averse partner **(B)** or a risk-neutral partner **(E)**, in the observation groups with a risk-averse partner **(C)** or a risk-neural opponent **(F)**, and in the competition groups with a risk-averse opponent **(D)** or a risk-neutral opponent **(G)**. The solid-colored curves denote the average endpoint, and the shaded areas indicate the standard error of the mean (s.e.m.). The dotted black lines and surrounding shaded areas denote the mean endpoint and s.e.m. of the virtual partner or opponent, respectively. The vertical dotted lines indicate the timing when the experimental session changed from the baseline to the main session. We compared the endpoint averaged in the baseline blocks (the shaded area from the 1st to 5th blocks) with that in the last five blocks in each group (the shaded area from the 13th to 17th blocks). The asterisks indicate *p* < 0.05.

The motor plan [i.e., true aim point μ in Equation (1)] of the virtual partner/opponent was determined based on the estimated movement variance σ^2^ in the past 40 trials for each participant. We prepared two properties: the risk-neutral (i.e., optimal) property and risk-averse property. For the first property, we set the true aim point at the optimal value for all 12 blocks. For the second property, we gradually changed the true aim point from optimal to risk-averse. In the first four blocks (i.e., 6th−9th blocks in Figure 2), the true aim point was set at the optimal aim point. In the next 4 blocks, the true aim point decreased in steps of 0.015 (i.e., 0.985μ^*^ at the 10th block, 0.97μ^*^ at the 11th block, 0.955μ^*^ at the 12th block, and 0.94μ^*^ at the 13th block). In the remaining four blocks, the aim point was set to 0.925μ^*^. After determining the true aim point, we generated the actual endpoint of the virtual partner or opponent by adding Gaussian noise, in which the variance was the same as the calculated movement variance σ^2^ in each participant. The average movement endpoints for these two properties are shown in Figure 2.

We did not add risk-seeking partners/opponents because they were likely to obtain 0 points; consequently, the participants could be deterred from following the task instructions (see Ota et al., 2020 for further details of manipulation of virtual opponent).

### Sample Size

The sample size was determined based on *a priori* power analysis using G^*^power (Faul et al., 2007). In our previous study (Ota et al., 2020), we found that a risk-seeking strategy taken at baseline was modulated to risk neutrality during competition with a risk-averse opponent. A comparison of the aim point (average endpoint) in the first 50 trials with that in the last 50 trials provided the effect size of *d* = 1.63. The *a priori* power analysis, with the paired *t*-test 0.05 significance criterion, 0.8 detection power, and 1.63 effect size, provided a sample size of 6 for each experimental group.

In our previous study (Ota et al., 2020), we ran four out of seven experimental groups: individual task, two competitive tasks, and observation task with a risk-averse partner. In those tasks, we chose the data from the first 6th participants who had participated in our previous study. For the remaining three tasks that had not been examined before running this study (observation with a risk-neutral partner and two cooperation tasks), we recruited 18 additional participants.

This study performed an *a priori* power analysis based on the modulation of the aim point rather than the modulation of risk sensitivity that was used for the *a priori* power analysis performed in our previous study (Ota et al., 2020). Because the evaluation of risk sensitivity required the past 40 trials, in calculating motor variability, the risk sensitivity for the first 40 trials in the baseline was not evaluated in this study. To fairly compare 50 trial data in the baseline and the main sessions, we performed *a priori* power analysis for the aim point. Notably, the following results were consistent with our earlier findings, regardless of the difference of the *a priori* power analysis.

### Statistical Analysis

Based on *a priori* analysis, we first examined whether the interaction types tested in this study affected the participant's aim point. We performed a paired *t*-test on the aim point (dependent variable) between the baseline session (1st−5th blocks) and main session (13th−17th blocks) (independent variable) for each group (see section Modulation of Average Endpoint Through Interaction). We then performed a paired *t*-test on the aim point (dependent variable) between the participant's actual data and the optimal aim point data (independent variable) for each group at two different time phases, baseline session and main session (see sections Risk-Seeking Property at the Baseline and Modulation of Risk Sensitivity). As the paired *t*-test was repeated for seven groups, Holm's correction was introduced to correct the statistical threshold. Since we estimated the optimal aim point given the past 40 trials, we included the endpoint data at the 5th block to examine whether the actual aim point was significantly different from the optimal aim point at baseline. For the same reason, we included the endpoint data across the 14th−17th blocks (i.e., the last 40 trials) to examine the difference in the actual and optimal aim point at the main session.

*p* < 0.05 was considered statistically significant. Cohen's *d* was calculated as an index of effect size. We use symbols *p*s and *d*s to denote multiple *p*-values (*p*) and Cohen's effect sizes (*d*), respectively, when multiple statistical tests are applied.

To investigate how the participant's aim point was modulated during three types of interactions, we fit a generalized linear model (GLM) on the relation between the participant's average endpoint data *A*_*s*_ and the virtual partner's/opponent's average endpoint data *A*_*v*_ (see section Influence of the Partners/Opponents on Motor Planning). Since we focused on how the partners or opponents affected the participant's aim point compared with the baseline, we subtracted the average endpoint at the baseline (1st−5th blocks) *A*_*i*_ from both measures of *A*_*v*_ and *A*_*s*_. Thus, the dependent variable *y* was *A*_*s*_ − *A*_*i*_, and the independent variable *x* was *A*_*v*_ − *A*_*i*_. For the same reason, we pooled the data in the risk-neutral and risk-averse groups within the same interaction type and fit the GLM to the pooled data for each type of interaction. In total, 144 endpoint data points (12 blocks × 6 participants × 2 properties) were included for GLM fitting in each interaction. We tested a constant model *y* = *b*_0_, a first-order equation model *y* = *b*_0_ + *b*_1_*x* and a second-order equation model $y={b_{0}+b}_{1}x+b_{2}x^{2}$, where *b*_0_ is an intercept (constant) and *b*_1_ and *b*_2_ are regression coefficients for the linear term and the quadratic term, respectively. For each interaction type, we chose the best model by adding the linear term and then the quadratic term and by examining whether the added regression coefficient was significant or not (Wald test).

## Results

### Modulation of Average Endpoint Through Interaction

Figure 2 indicates the arm-reaching movement endpoints averaged across all participants in each group. We compared the average endpoint across the 1st−5th blocks (baseline session) and that across the 13th−17th blocks (main session) (the shaded areas in Figure 2). The average endpoint significantly decreased during competition with a risk-averse opponent from the baseline blocks (Figure 2D, paired *t*-test, *p* = 0.018, *d* = 1.42). In the competition with the optimal opponent, there was a significant increase in the average endpoint from the baseline session to the main session (Figure 2G, paired *t*-test, *p* = 0.041, *d* = −1.11). There were no significant modulations in the average endpoint in the cooperative groups and observation groups from the baseline to the main session (Figures 2B,C,E,F; paired *t*-tests; *p*s > 0.24, *d*s < 0.39). Similarly, in the individual group, there was no significant difference (Figure 2A, paired *t*-test, *p* = 0.057, *d* = −0.24). The effect size observed in the competition with a risk-averse opponent was *d* = 1.42, and this effect was 3.6 times larger than the strongest effect size shown in the other five types of interaction (i.e., *d* = 0.39 in the observation with the risk-averse partner; median effect size was 0.01 among the other five interactions). In sum, both the individual situations and five types of interaction did not modulate the average endpoint as shown in the competition with a risk-averse opponent.

### Risk-Seeking Property at the Baseline

Although our results show the effect of virtual opponents on motor planning, the modulation of the aim point does not solely indicate that the participants made the optimal motor plan. Therefore, we focused on the risk sensitivity analysis below.

**Figure 3:**
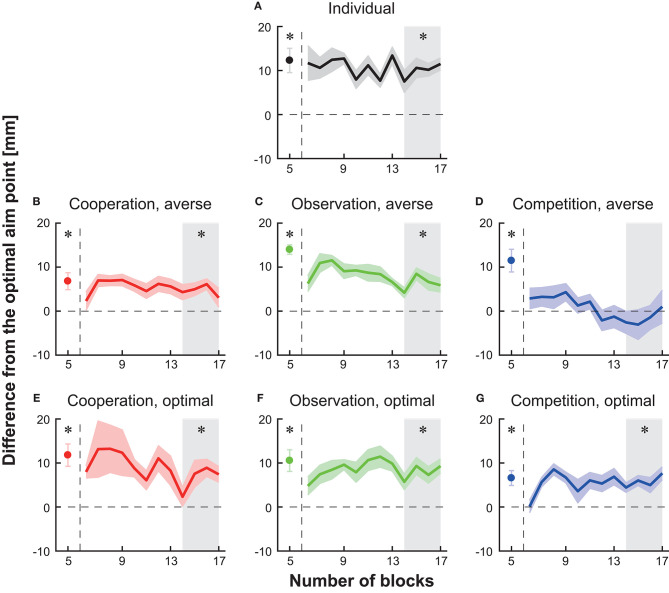
Block-to-block variation in the difference in average endpoint from the optimal aim point to the participant's aim point (i.e., risk-sensitivity). **(A)** Individual group **(B)** cooperation group with a risk-averse partner **(C)** observation group with a risk-averse partner **(D)** competition group with a risk-averse partner **(E)** cooperation group with a risk-neutral partner **(F)** observation group with a risk-neutral partner **(G)** competition group with a risk-neutral partner. A positive risk-sensitivity value denotes a risk-seeking strategy, whereas a negative value denotes a risk-averse strategy. The horizontal dotted line indicates the optimal and risk-neutral value (i.e., the difference is 0). The solid-colored curves indicate the averaged data across participants, and the shaded areas indicate the s.e.m. (6–17 blocks). The participant's average at the 5th baseline block is represented as a filled circle. The vertical dotted lines indicate the timing when the experimental session changed from the baseline to the main session. The asterisks denote a significant difference (*p* < 0.05) from the optimal aim point at the 5th baseline block or at the 14th–17th blocks (the gray-shaded area).

We first tested whether the participant's aim point was different from the optimal aim point in the individual setting. At the 5th block in the baseline session, the average endpoint was significantly larger than the optimal aim point in all seven groups (circles in Figures 3A–G, paired *t*-tests with Holm correction, *p*s < 0.023). When the participants in the individual group continued the individual task after the baseline, a significant deviation from the optimal aim point was still found in the last 40 trials in the main block (shaded area in Figure 3A, paired *t*-test with Holm's correction, *p* = 0.002). Therefore, the participants in all seven groups took a risk-seeking strategy at baseline, and the participants in the individual group persisted a risk-seeking strategy thereafter.

Moreover, we found a significant deviation from the optimal aim point in the last 40 trials (blocks 14th−17th) in five types of interaction except for competition with a risk-averse opponent (shaded area in Figures 3B,C,E–G, paired *t*-tests with Holm's correction, *p*s < 0.023). These results suggest that a risk-seeking property still exists during cooperation tasks, observation tasks, and competitive task with the optimal opponent.

### Modulation of Risk Sensitivity

We then tested a difference in the actual and optimal aim point in the 14th−17th blocks in the competition with a risk-averse opponent group and found no significant deviation from the optimal aim point [shaded area in Figure 3D, the difference from the optimal = −1.52 ± 2.92 (mean ± s.e.m.), paired *t*-test with Holm correction, *p* = 0.62]. Taken together, these results indicate that among the combinations tested in this study, competition with a risk-averse opponent reduced the participant's aim point from the baseline. Since the risk-sensitivity value at the competition was not significantly different from the risk-neutral value, optimal motor planning was induced.

### Influence of the Partners/Opponents on Motor Planning

**Figure 4:**
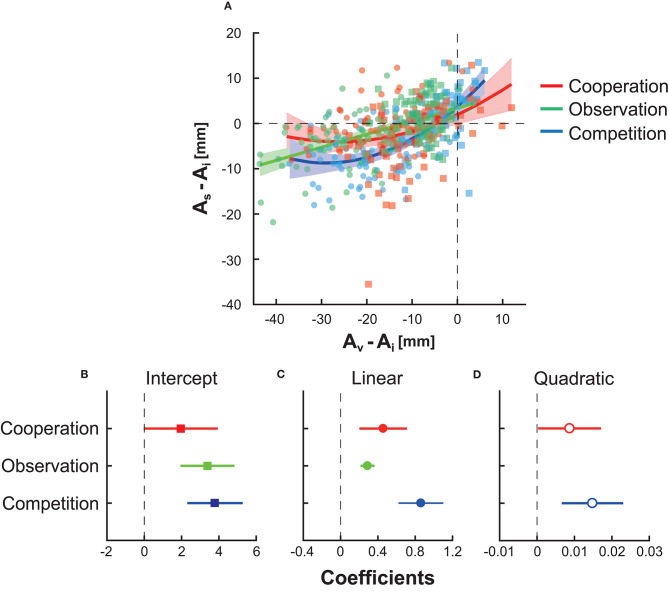
The influence of the virtual partner/opponent's motor plan on the participant's motor plan. **(A)** The horizontal and vertical axes indicate the virtual partner's or opponent's aim point *A*_*v*_ and participant's aim point *A*_*s*_ in each block, respectively. These values were subtracted from the participant's aim point at the baseline *A*_*i*_ to evaluate the influence relative to the baseline performance. Each rectangle indicates the data in risk-neutral groups, whereas each circle denotes the date in risk-averse groups. The second-order equation fit best in cooperation (red curve) and competition (blue curve), while the first-order equation fit best in observation (green line). Shaded areas around the solid lines indicate 95% confidence intervals of the fitting line or curves. **(B)** The fitted intercept and its 95% confidence interval. **(C)** The fitted regression coefficient for the linear term. **(D)** The fitted regression coefficient for the quadratic term.

Finally, we investigated how the virtual partner's/opponent's motor plan modulated the participant's motor plan (Ota et al., 2020). Because we assumed that block-to-block variation was more important to the average endpoint than to movement variability or risk sensitivity, this analysis focused on the average endpoint. Figure 4A shows the plots of the participant's average endpoint *A*_*s*_ against the virtual partner's/opponent's average endpoint *A*_*v*_, relative to the average endpoint at the baseline *A*_*i*_, for three types of interactions. A negative value on the *y*-axis (i.e., *A*_*s*_ − *A*_*i*_) indicates a decrease in the participant's endpoint from the baseline. A negative value on the *x*-axis (i.e., *A*_*v*_ − *A*_*i*_) indicates that the partners or opponents exhibit smaller endpoints than the participant's baseline endpoint.

We fit a general linear model (see section Statistical Analysis). Figures 4B–D show the fitted intercept, regression coefficient for the linear term, and that for the quadratic term. In competition (blue line, shaded area, or dots) and cooperation (red line, shaded area, or dots), the second-order equation model was selected (for competition, *R*^2^ = 0.460 *and p* = 5.31 × 10^−20^ from the constant model; for cooperation, *R*^2^ = 0.109 *and p* = 1.39 × 10^−4^ from the constant model). In observation (green line, shaded area, or dots), the first-order equation model was selected (*R*^2^ = 0.283 *and p* = 4.19 × 10^−12^ from the constant model). The second-order equation indicated the non-linear influence of the partner/opponent on the participants' motor plan. In contrast, observation has a linear influence in the current experimental setting. In Figure 4A, the upper bound of the 95% confidence interval in competition (blue-shaded area) was smaller than the lower bound of the interval in cooperation (red-shaded area) and observation (green-shaded area) when −30 ≤ *A*_*v*_ − *A*_*i*_ ≤ −10. This suggests that competition with risk-averse opponents had a larger inhibitory effect on the endpoint than the other types of interactions with risk-averse opponents. In summary, competition was more effective than cooperation or observation in inhibiting a risky motor plan.

We also fit a GLM on the modulation of the participant's aim point from the previous block. We used the difference in the aim points from the *t*th to *t* + 1th block, *A*_*s,t*+1_ − *A*_*s,t*_, as the dependent variable *y* and the aim point in the *t*th block, *A*_*s,t*_, as the independent variable *x*. In this GLM, we chose the same model selected above for each interaction type (second-order equation for competition and cooperation, first-order equation for observation) for a fair comparison. As a result, we found the *R*^2^ value of 0.160 in the competition. Compared with the *R*^2^ of 0.460 when fitting to the relation between the participant's and opponent's aim points, the fitting performance decreased. Therefore, the participants were likely to determine their strategy based on their opponent's behavior rather than on their own aim point in the previous block. In the cooperation groups, we found the *R*^2^ value of 0.351, which was a better fit than the fit in Figure 4 (*R*^2^ = 0.106). This indicates that the participants determined their aim points primarily based on their own aim point in the previous block with little reference to their partner's behavior. In the observation groups, we found the *R*^2^ value of 0.319, which was slightly better than the fit in Figure 4 (*R*^2^ = 0.283). Although the influence of cooperation and observation was supported by several findings (Richardson et al., 2007; Peng and Hsieh, 2012; Ganesh et al., 2014; Ikegami and Ganesh, 2014), the influence was not as large in motor planning in our decision-making task.

## Discussion

### Summary of Results

Previous studies have investigated the optimality of human motor planning. Although risk-averse or risk-neutral motor planning has been observed for several tasks (Trommershäuser et al., 2003, 2005; Nagengast et al., 2010; Onagawa et al., 2019) and there is an individual difference (see Nagengast et al., 2011b; Ota et al., 2016), humans generally demonstrate a risk-seeking motor planning strategy not only in a continuous motor decision task such as the one used in this study (Nagengast et al., 2011b; O'Brien and Ahmed, 2013; Ota et al., 2015, 2016, 2019b) but also in a two-alternative forced choice task (Wu et al., 2009; Nagengast et al., 2011a). A suboptimal motor plan is possibly caused by overconfidence and is also seen in real sports fields (Neiman and Loewenstein, 2011; Skinner, 2012). However, only a few studies have examined how to modify the risk-seeking tendency. The current study addressed this question using interpersonal interaction since interactions with others influence human actions (Richardson et al., 2007; Peng and Hsieh, 2012; Ganesh et al., 2014; Ikegami and Ganesh, 2014). We tested a competition, cooperation, and observation scenario with either risk-neutral or risk-averse opponents/partners. We confirmed that competition with a risk-averse opponent reduced the participant's aim point (Figure 2) and that the changed motor plan became risk-neutral (Figure 3). This outcome was explained by non-linear and inhibitory influences that emerge via competition (Figure 4). Our results confirm that competition with a risk-averse opponent has a larger influence than cooperation or observation in terms of modulating a motor plan under risk.

### Why Did the Motor Plan Approach the Optimal Point?

We have already excluded several possibilities for this question. The first possibility is that social facilitation induced by opponents (Zajonc, 1965) affects motor planning. If this were true, the same effect observed in competition should have been found in observation and cooperation. Second, the binary outcome (i.e., win or lose) affected motor planning. Our earlier study examined this possibility by setting a binary outcome condition in which participants were instructed to exceed the total score that was presented at the beginning of each block (Ota et al., 2020). The results showed that attempting to exceed the total score without an opponent does not change motor planning.

Our hypothesis is that the opponent's action has a non-linear and inhibitory influence on motor planning (Figure 4). When the opponents obtained a higher score than the participants (i.e., *A*_*v*_ − *A*_*i*_ ≥ 0), the participants sought a higher score than the baseline (i.e., *A*_*s*_ − *A*_*i*_ ≥ 0). When the opponents obtained a lower score than the participants (i.e., *A*_*v*_ − *A*_*i*_ ≤ 0), the participants sought a lower score than the baseline (i.e., *A*_*s*_ − *A*_*i*_ ≤ 0). These effects of the opponents can be considered synchronization in competitive tasks (Naber et al., 2013; Varlet and Richardson, 2015). However, if such synchronization were the factor, the participants should have exhibited a risk-averse strategy when their opponents were highly risk-averse (i.e., *A*_*s*_ − *A*_*i*_ ≤ −40). This was not the case in this study: the participants did not further decrease their aim point when their opponents were highly conservative.

Thus, we speculate that a win-stay lose-shift strategy (Nowak and Sigmund, 1993) was utilized along with the effect of the synchronization. That is, participants maintain the same strategy when they win the competition and switch the strategy when they lose. The participants would not decrease their aim point after their opponents began adopting highly conservative behavior, as the participants could already easily beat the opponent. There is no reason to adopt a win-stay lose-shift strategy in cooperation and observation tasks due to the nature of the tasks. Based on our earlier work (Ota et al., 2020) and this study, we now consider that the mixture effect between non-linearity (win-stay lose-shift) and inhibition (synchronization) is a possible candidate for why motor planning was optimized. Indeed, a previous study proposed a preliminary model that predicted optimal motor planning without knowledge of motor variability but by a shift in an aim point in response to having a negative outcome (Brenner and Smeets, 2011).

If the participants used a win-stay lose-shift strategy, one might consider when they predicted their opponent's score and determined their strategy. We consider three possibilities for this question as follows: before each experimental block, during the block, and both before and during the block. Trial-to-trial variability can be a problematic and noisy factor in attempting to clarify which of the three possibilities is at play. One possible way to estimate the unobservable true aim point and its fluctuation is to use some variants of the state space model (Ghahramani and Hinton, 2000; Takiyama et al., 2009; Takiyama and Okada, 2011; Naruse et al., 2013). For example, if the true aim point is invariant within each block and varies between blocks, it is plausible that the strategy is determined before each block on the basis of the prior history of the opponent's total scores. If, on the other hand, the true aim point varies within each block and is invariant between blocks, the strategy is likely determined during the block based on upcoming information on the opponent's score in each trial.

### Relevance to Previous Literature in Motor Control and Learning

In general, there are (at least) three stages in human movement: the planning stage to make a movement plan, the control stage to control a movement as planned, and the learning stage to acquire the internal model and update a motor plan given an error. In the long history of motor control and learning literature, the target (i.e., motor plan) has been visually guided in most cases (Flash and Hogan, 1985; Uno et al., 1989; Lackner and Dizio, 1994; Shadmehr and Mussa-Ivaldi, 1994; Harris and Wolpert, 1998; Todorov and Jordan, 2002; Takiyama et al., 2015). It has been shown that humans optimally control an arm-reaching movement to the given target (Flash and Hogan, 1985; Uno et al., 1989; Harris and Wolpert, 1998; Todorov and Jordan, 2002). Humans also adapt to environmental changes such as mechanical perturbation (Lackner and Dizio, 1994; Shadmehr and Mussa-Ivaldi, 1994) or visuomotor transformation (Takiyama et al., 2015). The problem lies in the planning stage, where ones need to decide on a target by themselves. In this stage, previous findings show a risk-seeking tendency (Wu et al., 2009; Nagengast et al., 2011a,b; O'Brien and Ahmed, 2013; Ota et al., 2015, 2016). The current study added a new finding regarding how three types of interpersonal interactions modulate a risk-seeking tendency.

Although observing another person who is learning to reach in a novel environment enhances motor learning (Mattar and Gribble, 2005; Malfait et al., 2010; McGregor et al., 2016), observing the performance of a risk-neutral virtual partner did not facilitate a risk-neutral motor plan. A lack of benefit of the observation in motor planning may or may not reflect a lack of engagement in the neural network engaged in observation (Malfait et al., 2010; McGregor et al., 2016) due to the virtual partner.

## Limitations

In our study, we defined the optimal aim point as the one for maximizing one's own expected gain. This may not be optimal in terms of maximizing the chance of winning to the opponents. However, our simulation showed that the difference in these two aim points is marginal, at least in our experimental setting (Supplementary Figure 1). We found that two aim points overlap when the opponent is nearly risk-neutral. The difference was shown only when the opponents were highly conservative, and the maximization of the chance of winning slightly shifted the aim point lower than the maximization of expected gain. Therefore, the participant's strategy might be riskier than the one maximizing the chance of winning. Since the opponent's aim point is unobservable in an actual experiment, the participants might shift their aim point toward the maximization of expected gain (see Supplementary Material for further discussion on this issue).

As for another limitation, it is still unclear whether our results are invariant in human–human interactions. Since most of the participants demonstrate risk-seeking behavior, we used virtual partners/opponents whose risk sensitivity and movement accuracy can be arbitrarily manipulated. Validating our findings in interactions with human participants is promising for future research.

## Conclusion

This study examined the effects of three types of interpersonal interactions on human motor planning under risk. We demonstrate that competition with a risk-neural opponent increases the participant's aim point from the baseline, whereas competition with a risk-averse opponent decreases the aim point. There were fewer effects in the modulation of motor plans during cooperation with risk-neutral/risk-averse partners and observation with those partners. Among the interaction types tested in this study, only competition with a risk-averse opponent leads to the optimal motor plan.

These results provide rich practical implications for decision-making training programs in sports and e-sports. That is, our results suggest that it is not always good to compete with strong (i.e., risk-neural and optimal) opponents. To improve a suboptimal and risk-seeking strategy in real sports fields [e.g., a shot selection problem in basketball players in NBA (Neiman and Loewenstein, 2011; Skinner, 2012)], one might need competition with various types of opponents, especially with more conservative and weaker opponents than them. Such training may provide athletes with a clue on what decision strategy should be taken. Our results also show behavioral evidence that humans flexibly change their decision strategy depending on the level (strength) of their virtual opponents (Figure 4). Therefore, this work highlights the importance of adjusting the level of computer opponents to improve performance in e-sports athletes. We hope that the results of this work contribute to the further development of decision-making training programs in sports and e-sports.

## Data Availability Statement

The raw data supporting the conclusions of this article will be made available by the authors, without undue reservation.

## Ethics Statement

The studies involving human participants were reviewed and approved by the ethics committees of the Tokyo University of Agriculture and Technology. The patients/participants provided their written informed consent to participate in this study.

## Author Contributions

KO and KT conceived and designed the experiments and wrote the manuscript. MT and KO performed the experiments. MT, KO, and KT analyzed the data and interpreted the results, revised the manuscript, and approved the final manuscript. All authors contributed to the article and approved the submitted version.

## Conflict of Interest

The authors declare that the research was conducted in the absence of any commercial or financial relationships that could be construed as a potential conflict of interest.
