# Supplementary material for: Competition Rather Than Observation and Cooperation Facilitates Optimal Motor Planning
Source: Front Sports Act Living. 2021 Feb 26;3:637225. doi: 10.3389/fspor.2021.637225 (PMC7959757; doi:10.3389/fspor.2021.637225)
Supplement: Supplementary file 1 [file Data_Sheet_1.pdf]

Competition rather than observation and cooperation facilitates optimal motor planning

Mamoru Tanae[1†], Keiji Ota[1,2,3†], Ken Takiyama[1]

[1]Department of Electrical and Electronic Engineering, Tokyo University of Agriculture and Technology, Tokyo, Japan.

[2]Department of Psychology, New York University, New York, United States.

[3]Center for Neural Science, New York University, New York, United States.

[†]These authors have contributed equally to this work.

Correspondence should be addressed to K.O. (keiji.ota@nyu.edu) or K.T. (t.j.ken.takiyama@gmail.com).

Analytical solution of expected gain

With a piecewise linear function as used in the main text, the expected gain (G) with Gaussian distribution

$$\mathrm{G}(\mu) = \int_{-\infty}^{\infty} f(x)\, \mathcal{N}(x|\mu, \sigma^2)\, \mathrm{dx}\ , \tag{1}$$

is analytically tractable when $\mathrm{f}(x) = a_1 x + a_0\ (x_0 \le x \le x_1)$ and $\mathrm{f}(x) = 0$ otherwise; $\mathrm{G}(\mu) = \int_{-\infty}^{\infty} \mathrm{f}(x)\mathcal{N}(x|\mu, \sigma^2)\, dx = \int_{x_0}^{\infty}(a_1 x + a_0)\mathcal{N}(x|\mu, \sigma^2)dx - \int_{x_1}^{\infty}(a_1 x + a_0)\mathcal{N}(x|\mu, \sigma^2)\, dx$. By using the Q-function $Q(x) = \int_{z}^{\infty} \mathrm{d}z\, \mathcal{N}(z|0,1)$, the expected gain can be written as $\mathrm{G}(\mu) = (a_1\mu + a_0)\left(Q\left(\frac{x_0-\mu}{\sigma}\right) - Q\left(\frac{x_1-\mu}{\sigma}\right)\right) + \frac{a_1\sigma}{\sqrt{2\pi}}\left(\exp\left(-\frac{(x_0-\mu)^2}{2\sigma^2}\right) - \exp\left(-\frac{(x_1-\mu)^2}{2\sigma^2}\right)\right)$. In a general form of f(x), the expected gain is analytically intractable, and either a numerical integral or Monte Carlo integral is useful for calculating the expected gain.

Analytical solution for variability of expected gain

With a piecewise linear function as used in the main text, the squared expected gain (G) with Gaussian distribution

$$G^2(\mu) = \int_{-\infty}^{\infty} f^2(x)\, \mathcal{N}(x|\mu, \sigma^2)\, \mathrm{dx}\ , \tag{2}$$

is analytically tractable when $\mathrm{f}(x) = a_1 x + a_0\ (x_0 \le x \le x_1)$ and $\mathrm{f}(x) = 0$ otherwise. By using the Q-function $Q(x) = \int_{z}^{\infty} \mathrm{d}z\, \mathcal{N}(z|0,1)$, the squared expected gain can be written as $G^2(\mu) = (a_1^2(\sigma^2 + \mu^2) + 2a_1 a_0\mu + a_0^2)\left(Q\left(\frac{x_0-\mu}{\sigma}\right) - Q\left(\frac{x_1-\mu}{\sigma}\right)\right) + \frac{2a_1^2\mu\sigma + 2a_1a_0\sigma}{\sqrt{2\pi}}\left(\exp\left(-\frac{(x_0-\mu)^2}{2\sigma^2}\right) - \exp\left(-\frac{(x_1-\mu)^2}{2\sigma^2}\right)\right) + \frac{a_1^2\sigma^2}{\sqrt{2\pi}}\left(\frac{x_0-\mu}{\sigma}\exp\left(-\frac{(x_0-\mu)^2}{2\sigma^2}\right) - \frac{x_1-\mu}{\sigma}\exp\left(-\frac{(x_1-\mu)^2}{2\sigma^2}\right)\right)$.

By using Eqs. (1) and (2), the variance of the expected gain can be written as

$$Var[G(\mu)] = G^2(\mu) - [G(\mu)]^2. \tag{3}$$

Distance between two expected gain distributions

To find the optimal aim point in the competitive task, we computed the distance between the expected gain distribution for a player's aim point and that for an opponent's aim point using Eqs. (1) and (3). We used d' for a measure of distance including uncertainty, which can be written as

$$d' = \frac{G(\mu_p) - G(\mu_o)}{\left(Var[G(\mu_p)]^{\frac{1}{2}} + Var[G(\mu_o)]^{\frac{1}{2}}\right)/2} \tag{4}$$

where μ_p is a player's aim point, μ_o is an opponent's aim point, and an unequal variance is assumed. A larger d' is a larger difference in the expected gain under the existence of uncertainty for two aim points. Therefore, the aim point to achieve the largest d' corresponds to the aim point to maximize the winning rate in the competitive task. We computed d' values by varying μ_p and μ_o (Suppl. Fig. 1) with a fixed motor noise σ at 15 mm. A cyan curve indicates the player's aim point that maximizes d' against each opponent's aim point. A red line indicates the optimal aim point μ^* computed by maximizing Eq. (1). A right dashed vertical line denotes the risk-neutral opponent's aim point used in our experimental setting (i.e., μ^*), whereas a left dashed vertical line denotes the most conservative (risk-averse) opponent's aim point (i.e., $0.925\mu^*$).

The simulation indicates that the aim point maximizing d' matches the aim point maximizing the expected gain when the opponent is close to risk-neutral (i.e., around the right dotted black vertical line in Suppl. Fig. 1), meaning that the maximization of expected gain is also the optimal strategy in competition with a risk-neutral opponent. When the opponent is highly risk-averse (i.e., around the left dotted black vertical line in Suppl. Fig. 1), the aim point maximizing d' shifts lower than μ^*, meaning that a shift to the risk-averse direction from the maximization of expected gain is optimal in terms of winning rate.

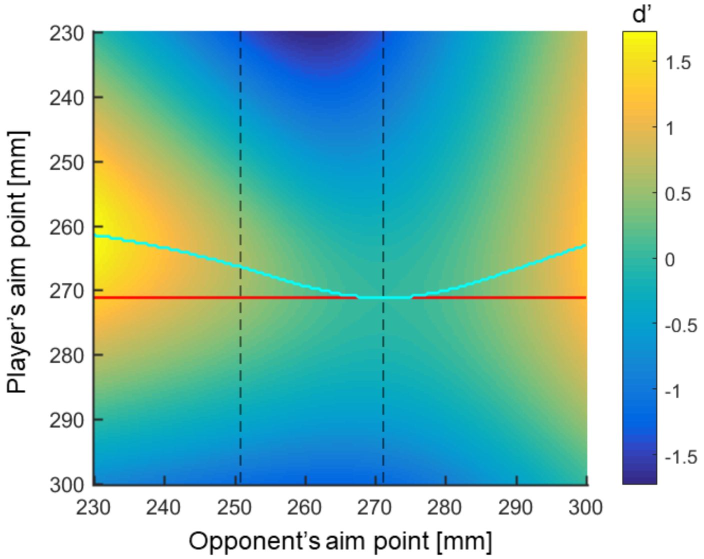

Supplementary Figure 1. Computed distance between the expected gain distribution for the player's aim point and that for the opponent's aim point.

In contrast to these optimal aim points in the competitive tasks, we found a risk-seeking tendency when the opponent was risk-neutral and a risk-neutral tendency when the opponent was risk-averse. There were at least possibilities inherent in our results. The first possibility is that the participant's strategy was in effect riskier than the one maximizing the chance of winning against any opponent. When the opponents were risk-neutral, the participants aimed closer to the penalty boundary than these two identical optimal aim points

(Figs. 2g and 3g). When the opponents were risk-averse, the participants approached risk-neutral (Figs. 2d and 3d), but in terms of chance of winning, they should have aimed slightly lower (Suppl. Fig. 1). That is, participants might demonstrate an overall risk-seeking tendency than the strategy maximizing the chance of winning. However, this hypothesis and simulation are based on the assumption that the opponent's aim point is fully known. In the actual experiment, the opponent's aim point and expected gain had to be estimated. Therefore, as the second possibility, it is considered that uncertainty in the opponent's aim point affected the participant's aim point. When the knowledge of the opponent's aim point is partially given and players need to estimate the opponent's aim point, a difference between a cyan curve and a red line would become small. It is obvious that the maximization of one's own expected gain is the only choice for maximizing the winning rate when the opponent's aim point is completely unknown. Thus, the overall shift from the maximization of the chance of winning can be considered the outcome that the participants geared towards the maximization of expected gain under a partially observable situation.
